# Gleanings from Our Exchanges

**Published:** 1874-04-01

**Authors:** 


THE INFLUENCE OF POSTURE ON "PRESYSTOLIC" CARDIAC MURMURS.—The influence of posture in altering or removing cardiac murmurs, forms the subject of a valuable paper by W. R. Gowers, M.D., in *The Practitioner* for December, 1873. Dr. Gowers states that several cases have come under his notice in which, after a careful and skilled examination in the erect posture, a heart has been declared free from murmur, when, had the patient been made to lie down, a bruit would have been heard, which could not have been overlooked. He believes that, in most instances, the murmur which precedes the first sound, and is commonly regarded as characteristic of mitral obstruction, is both louder and longer in the recumbent than in the erect posture, and that in many instances, more frequently than in the case of any other cardiac murmur due to an organic cause, it may be heard in one posture and not in another—may be loud when the patient is lying down, and inaudible when he is standing up.—*Boston Medical and Surgical Journal.*

---

DEATH FROM INHALATION OF THE PRODUCTS OF COMBUSTION IN AN OPEN FIREPLACE WITH A CHIMNEY.—A man and his wife, both strong and healthy, went to bed in a room in the fireplace of which a fire had been lighted shortly previous. During the night, the woman awoke with symptoms of suffocation—arose, but staggered about, and fell to the floor. The husband rose to aid her; tried to light the gas, and failed, but likewise fell to the floor unconscious. When discovered in the morning, the man was dead; while the woman was so far gone that she was with difficulty resuscitated. The theory with regard to the death was, that the night being a very stormy one, and the fire small, the wind, blowing sharp and steady over a low chimney, acted as a damper, and effectually prevented the gases from making their escape by the way provided for them.—*Edinburgh Medical Journal*, January, 1874.

---

NEURALGIA IN INFANTS.—Children from two to six weeks old, especially males, suffer frequently with attacks of pain in the bowels, coming on about midnight, and lasting until four or five in the morning. Children thus affected cry violently, but towards morning become quiet, fall asleep, and the next day are well as ever. This enteralgia does not seem to be caused by any fæcal accumulations; it is very noticeable, however, that during the paroxysm they pass no water, and at the end of it a large quantity of pale-colored urine comes away, as after an hysterical attack. The cause of this retention of urine is unknown. The disease affects children of all classes of society, indiscriminately, without reference to their hygienic condition. The remedy recommended by Dr. Boyd (*Edinburgh Medical Journal*, Feb., 1873; *Schmidt's Jahrbucher*, 1873, No. 2) is spiritus ætheris nitrosi, eight or ten drops in a drachm of water. Immediately after, with escape of wind and the passage of a considerable quantity of urine, the crying ceases, and the little patient goes to sleep.—*Boston Med. and Surg. Journal.*

---

VIENNA is now supplied with clear spring water, at a temperature of 50 degrees F., brought from the heights of the Sœmmering, a distance of about seventy miles.

